# Dishwashing liquids with nuclease and protease: An improved biocompatible solution for the removal of adherent bacteria from fruits and vegetables

**DOI:** 10.3934/microbiol.2025048

**Published:** 2025-12-29

**Authors:** Lyudmila Ayzatullina, Sofia Kolyshkina, Elizaveta Patronova, Viktor Filatov, Iva Zadorina, Maya Kharitonova, Mikhail Bogachev, Airat Kayumov

**Affiliations:** 1 Institute of Fundamental Medicine and Biology, Kazan Federal University, Kazan, Russian Federation; 2 Institute of Biomedical Systems and Biotechnology, Peter the Great St.Petersburg Polytechnic University, Saint Petersburg, Russian Federation; 3 Department of Pharmaceutical Chemistry and Organization of Pharmaceutical Business, Faculty of Fundamental Medicine, Lomonosov Moscow State University, Moscow, Russian Federation; 4 Biomedical Engineering Research Centre, St. Petersburg Electrotechnical University, St. Petersburg, Russian Federation

**Keywords:** dishwashing liquids, enzymes, nuclease, protease, biofilm, adherent cells, bacteria removal, vegetables peels and leaves

## Abstract

While plant food is an obligate part of human nutrition, vegetables and fruits are often contaminated by adherent foodborne pathogens, in turn requiring biocompatible solutions for their efficient elimination. We report the effect of proteinase (subtilisin) and nuclease (DNAse) additions to the dishwashing liquid for a more efficient removal of adherent bacteria and biofilms from glass surfaces and vegetables. The 15 min treatment with solely 0.06% protease solution decreased preformed biofilms of *S. aureus* and *S*. Typhimurium threefold, and treatment with 0.25% nuclease reduced them twofold, respectively. While nuclease itself was of low efficiency, the protease-nuclease mixture (0.06% of each protein) reduced the biomasses of biofilms of these bacteria fourfold, as well as biofilms of *E. faecalis*, *E. coli*, and *K. pneumoniae* twofold. The addition of enzymes to the dishwashing liquid increased the removal of Gram-negative bacteria from the glass 5–10-fold compared to basic liquid. Furthermore, enzymes enhanced the removal of adherent bacteria from lettuce, cucumber, celery, and apple up to 100-fold for *S. aureus* and *E. faecalis* and 20-fold for Gram-negative species, respectively, compared to the basic dishwashing liquid, as indicated by CFUs count and qPCR data. These data suggest that protease, both individually and especially in mixture with nuclease, is an attractive additive to dishwashing liquids to provide the removal of up to 99% of adherent bacteria from dishes, fruits, and vegetables.

## 1. Introduction

Plant food is an obligate part of the human diet, with a recommended consumption of 400 g per day per capita of unprocessed fruits and vegetables, according to World Health Organization (WHO). However, surfaces of fruits and vegetables represent natural habitats for many bacteria, including those responsible for the emergence of foodborne diseases [1]–[3]. Harvesting, processing, and transport movements are the main post-harvesting contamination sources [1],[4],[5]. According to the European Food Safety Authority (EFSA) and the European Centre for Disease Prevention and Control (ECDC), bacterial food contamination is the primary cause of foodborne disease outbreaks, which exhibited a stunning 44% increase in 2022 compared to 2011 [6].

The list of food-borne bacteria most frequently linked to the fresh produce-related outbreaks includes, although is not limited to, *Salmonella spp*., *Listeria monocytogenes*, *Staphylococcus aureus*, *Campylobacter spp*., *Escherichia coli*, and *Shigella*, depending on the type of plant, growing conditions, transporting, and storage [7],[8]. *Klebsiella pneumoniae* and *Enterococcus faecalis* can also contaminate fresh products, increasing the risk of contracting infections [9],[10]. Due to differences in the structure and chemical composition of fruits and vegetables, they carry different infectious potential. From a global perspective, WHO categorized lettuce and salads (all varieties), leafy vegetables (spinach, cabbage), and fresh herbs as the highest priority in terms of fresh produce safety [7],[11]. In addition, cucumber has been identified as a novel vehicle of *Salmonella* and linked to outbreaks since 2013 [8]. Fresh apple products, including juices, were linked to outbreaks caused by *E. coli* O157:H7 [12].

While compliance with hygienic procedures is generally believed to significantly reduce the infection risks, opposite data are reported. Thus, it was previously shown that rinsing vegetables and fruits under cold tap water reduces *Listeria innocua* within the range of 1.41 to 2.10 log CFU/g, which even exceeds the effect of chlorinated water (50 to 200 ppm), the most widely used decontaminant in the food industry, and gaseous sanitizers [13],[14]. On the other hand, many bacteria remain adhered to the surfaces of plants and vegetables, thus increasing the risk of a food-borne infection (for a recent review, we refer to [15]. Accordingly, more research is needed to come up with a universal and biocompatible solution for fruit and vegetable washing.

A potential risk of contamination is also associated with strictly adherent bacterial cells, as well as those embedded into biofilms on the surfaces of vegetables and fruits, enhancing their survival in harsh environments and resisting antimicrobial treatment [16]–[20]. Microbial biofilms consist of extracellular polymeric substance (EPS), which is composed of extracellular proteins, lipids, nucleic acids (extracellular DNA and extracellular RNA), polysaccharides, and secondary metabolites [21]. Proteins, as the major component of EPS of most Gram-positive bacteria, are responsible for cell aggregation, surface adhesion, and structural integrity of the biofilm. Therefore, their destruction is believed to be one of the most effective ways to eliminate biofilms. Extracellular DNA is another vital component of EPS, facilitating microbial adhesion, cell signaling, gene transfer, and biofilm matrix stabilization. Targeting these two vital components of EPS can lead to its degradation and detachment of bacteria [21]–[23].

To date, a number of approaches to the degradation of matured biofilms have been proposed. One of the strategies is to employ various hydrolytic enzymes to digest EPS components, thus disrupting the biofilm integrity. Enzymatic treatment possesses several benefits, including high effectiveness under low concentrations, low risk of antibiotic resistance development, and biodegradability, while targeting both growing and pre-existing biofilms simultaneously [21]. Thus, several enzymes have been offered as antibiofilm agents. In particular, repression of the biofilm formation by *Escherichia coli* by subtilisin A [24], degradation of a protein component of staphylococcal biofilms by chymotrypsin [25], and inhibition of the biofilm formation of *P. aeruginosa*, *L. monocytogenes*, and *S. epidermidis* by serratopeptidase from *Serratia marcescens* [26],[27]. The plant cysteine proteases ficin, papain, and bromelain have been shown to efficiently degrade the matrix of biofilms formed by *S. aureus*, *S. epidermidis*, and *M. luteus*, all including a substantial fraction of proteins [28]–[30]. Additionally, DNase was reported as an efficient antibiofilm tool [31]–[35]. Furthermore, combinations of enzymes have been offered to improve biofilm destruction. Thus, DNase I and proteinase K were tested to induce the dispersal of pre-existing biofilms of *Listeria monocytogenes* and multi-species oral biofilms [36]–[38]. Nevertheless, most of the mentioned approaches were offered for medical and veterinary use. Only a few investigations considered the potential of enzymes to facilitate the removal of foodborne pathogens from the surfaces of fruits and vegetables in daily household use [39].

While enzymes in laundry detergents and household cleaners are widely used to remove organic contaminations, thus significantly enhancing the performance in environmentally safe mode [40]–[42], almost no quantitative data regarding pathogens removal are available to date. In this work, a commercially available protease (subtilisin, EC number 232-752-2) and deoxyribonuclease (CAS number 232-667-0) alone and in composition with dishwashing solution were tested for the biofilm degradation. As well, we have quantified the elimination of adherent cells of food-borne bacteria *Salmonella* Typhimurium, *Klebsiella pneumoniae*, *Escherichia coli*, *Staphylococcus aureus*, and *Enterococcus faecalis* from various fruits and vegetables (lettuce, cucumber, celery, and apple) by base dishwashing solution and those containing the enzymes and demonstrate for the latter up to 100-fold better removal of *S. aureus* and *E. faecalis* and 20-fold for Gram-negative species.

## 2. Materials and methods

### 2.1. Dishwashing liquids and enzymes

The composition of the base dishwashing liquid is present in Table S1. Additionally, the deoxyribonuclease (CAS 9003-98-9), protease (subtilisin, CAS 9014-01-1), or both enzymes were added to testing dishwashing liquid samples to final concentrations as indicated in Tables S2–S4.

### 2.2. Strains and growth conditions

*Staphylococcus aureus subsp. aureus* ATCC 29213, *Enterococcus faecalis* ATCC 19433, *Escherichia coli* ATCC 25922, *Salmonella enterica* subsp. *Enterica* serovar Typhimurium ATCC 14028, and *Klebsiella pneumoniae subsp. pneumoniae* ATCC 13883 were used in the study. Bacteria were maintained and grown in the LB broth. To establish the biofilm, basal medium (BM broth, glucose 5 g, peptone 7 g, MgSO₄ × 7H₂O 2.0 g, and CaCl₂ × 2H₂O 0.05 g in 1.0 liter of tap water) has been used [43].

### 2.3. Biofilm assays

Bacterial biofilms were established by inoculating bacterial suspension (2–9×10^6^ CFU/mL) in BM broth in 24-well TC-treated polystyrene plates (1 mL per well), followed by 48 h incubation under static conditions at 37 °C. Then the broth was removed, and wells were filled with the samples of nuclease, protease, or their mixture in either phosphate-buffered saline (PBS, pH 7.5) or dishwashing base at concentrations as indicated, and the incubation was continued for the next 15 min. Then, the liquid was discarded, and the wells were gently washed several times with PBS and subjected to the crystal violet staining assay as described previously [44]. The 2-fold reduction of the residual dye absorption has been considered a significant reduction of the biofilm.

### 2.4. Bacterial adhesion on glass Petri dishes

The 90-mm glass Petri dishes were used in experiments with bacterial adhesion on their surface and following washing by the dishwashing liquids. Briefly, sterile Petri dishes were incubated with a bacterial suspension (density of 10^6^ CFU/mL) for 2 hours at room temperature for bacterial adhesion to the surface. Then the dishes were rinsed with sterile distilled water to remove non-adherent cells and filled with either sterile distilled water, dishwashing liquid with enzymes, or the dishwashing liquid without enzymes as a control. After 15 min of incubation, dishes were rinsed in sterile water, and adherent bacteria were harvested by swabbing the surface. Swabs were placed in 2 mL tubes and resuspended in 0.2 mL of sterile 0.9% NaCl solution, and the cells were quantified by preparing a series of 10-fold dilutions in 3 technical repeats and dropping of 5 µL onto LB agar plates. CFUs were counted from the two last drops containing 5-15 colonies and further averaged [45],[46]. Alternatively, total DNA was isolated from the remaining bacterial suspension, and quantitative PCR was performed.

### 2.5. Bacterial adhesion on vegetables and count of viable bacteria

Iceberg lettuce, celery, cucumber, and the red apple were used in experiments of bacteria adhesion on their surface and following washing by the dishwashing liquids. Briefly, round fragments with a diameter of 1.5 cm were cut from the lettuce and celery leaves or cucumber and the red apple peels, immersed into a bacterial suspension with a density of 10^6^ CFU/mL and incubated for 2 hours at room temperature for bacterial adhesion to the surface. Then the plant samples were rinsed in sterile distilled water to remove non-adherent cells and placed in either dishwashing liquid with enzymes, sterile distilled water, or the dishwashing liquid without enzymes as a control. After 15 min of incubation, samples were rinsed in sterile water and placed in 2 mL tubes containing 0.2 mL of sterile 0.9% NaCl solution, and the cells were washed off by vigorous shaking in an MP BIO FastPrep homogenizer. To count the cells that remained adherent, the washed leaf or peel samples were disrupted in tubes with 0.1 mm glass beads by MP Bio FastPrep homogenizer (2 × 20 sec, speed 4.0). The residual plant material and glass beads were removed by centrifugation (2 min at 4000 rpm), and the supernatant was used to prepare a series of 10-fold dilutions in 3 technical repeats and dropped by 5 µL onto LB agar plates. CFUs were counted from the two last drops containing 5–15 colonies and further averaged [45],[46]. Alternatively, total DNA was isolated from the remaining bacterial suspension, and quantitative real-time PCR was performed.

### 2.6. DNA extraction and quantitative PCR

For the quantitative PCR-based assessment of adherent cell amount, 200 µL of supernatant obtained in the previous step was subjected to DNA extraction by DNA extraction kit (DU-250, Biolabmix®, Russia) followed by quantitative PCR with HS-qPCR SYBR Blue kit (Biolabmix®) under conditions recommended by the manufacturer with SYBR Blue detection. The following oligonucleotides were used: 16S–dir: 5′-GGGACCCGCACAAGCGGTGG-3′, 16S–rev: 5′-GGGTTGCGCTCGTTGCGGGA-3′. Data were expressed as DNA copy changes, folded as 2^(Ct1-Ct0)^, where Ct0 was determined for samples before washing with dishwashing liquid and Ct1 was determined for samples after a 15-minute wash with dishwashing liquid with a subsequent rinse in pure water.

### 2.7. Statistics

Experiments were carried out in biological triplicates with three technical repeats in each one. The statistical significance of the results was assessed using either a Multiple t-test with Holm-Sidak correction for data with Gaussian distribution or a Kruskal-Wallis ANOVA test with Dunn's post-hoc correction with the significance threshold at p < 0.05.

## 3. Results

### 3.1. The effect of nuclease and protease on biofilms in vitro

To evaluate the biofilm destruction potential of protease, nuclease, and their mixture, *S. aureus*, *E. faecalis*, *E. coli*, *S*. Typhimurium, and *K. pneumoniae* were grown in BM broth for 48 h on 24-well TC-treated plates. Next, the plates were washed twice with PBS 7.4, followed by incubation with either protease or nuclease (0.06, 0.12, or 0.25%) or the protease-nuclease mixture (0.06, 0.12, and 0.25% of each enzyme) in PBS with pH 7.0. The incubation time was set up to 15 min, representing typical times of dishes and vegetables exposure to dishwashing liquids in common household applications. Then, the culture liquid was discarded, and residual biofilms were quantified with crystal violet staining.

**Figure 1.**
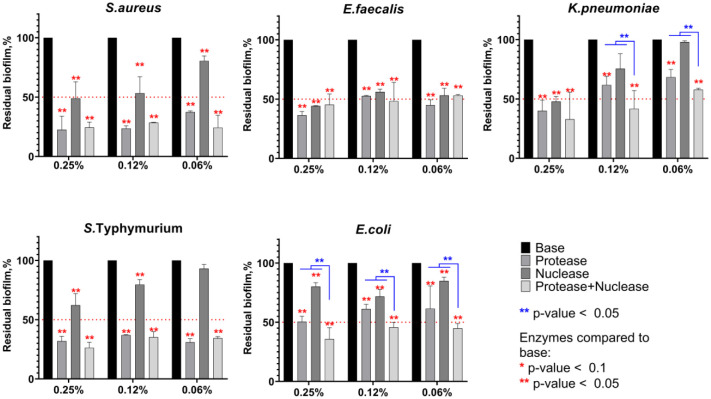
The destruction of biofilms of *S. aureus*, *E. faecalis*, *E. coli*, *S*. Typhimurium, and *K. pneumoniae* by either protease or nuclease (0.06, 0.12, and 0.25%) or the protease-nuclease mixture (0.06, 0.12, and 0.25% of each protein) in PBS with pH 7.0. 48-hour-old biofilms were treated for 15 min with enzymes, and residual biofilms were quantified by CV staining. Averages and SDs are shown. The significance of differences between treated and untreated samples was assessed using the multiple t-test with Holm-Sidak correction.

After 15 minutes of treatment, a clear dose-dependent reduction of biofilm biomass could be observed for all bacteria. The protease led to a three-fold reduction of biofilm biomass in the case of *S. aureus*, *E. faecalis*, and *S*. Typhimurium at a concentration of 0.06%, and up to a four-fold reduction at a concentration of 0.25% (Figure 1). For biofilms of *E. coli*, *E. faecalis*, and *K. pneumoniae*, 70–80% of the residual biomass was retained after treatment with 0.06% protease solution, while a two-fold reduction of the biomass was achieved at a concentration of 0.25%, apparently because of the prevalence of polysaccharides in their biofilm matrix [47]. The nuclease was less efficient and could provide destruction of biofilms of *S. aureus*, *E. faecalis*, and *K. pneumoniae* by one-half only at a concentration of 0.25%. The protease-nuclease mixture demonstrated higher effectiveness compared to pure enzymes against *E. coli* and *K. pneumoniae*, as significant difference could be observed between treatment with solely either protease or nuclease and their mixture (at 0.06 and 0.12%). Thus, at a concentration of both enzymes of 0.06%, a two-fold destruction of biofilms formed by either *E. coli* or *K. pneumoniae* could be achieved compared to 70-80% of residual biofilms after treatment with pure enzymes. In the case of *S. aureus*, *E. faecalis*, and *K. pneumoniae*, the biofilm-destruction rate remained similar for almost all tested concentrations of enzymes mixture and was slightly higher compared to protease treatment, although not significantly. Taken together, these data demonstrate the advantage of combined enzymatic treatment to obtain a more universal antibiofilm composition.

As the pH value of the base dishwashing liquid varies in the range of 5.0–6.0 (see Tables S1–S4), we tested the biofilm destruction by these enzymes in PBS with pH 6.0 and pH 5.0 (Figures S1 and S2). As one can see from the figures, the biofilm destruction effectiveness by protease reduced significantly with the pH drop. By contrast, the protease-nuclease mixture retained the ability to reduce the biofilm biomasses of all bacteria, although only a half-reduction of the biomass could be achieved after 15 min of treatment at pH 6.0 and 70–80% of the biofilm retained after treatment at pH 5.0. Nevertheless, a treatment with protease-nuclease mixture provided significantly more effective removal of *E. coli* and *K. pneumoniae* biofilms compared to solely enzymes at concentrations of 0.06%; compared to solely protease, the mixture was more effective at any concentrations. In the case of *S. aureus* and *S*. Typhimurium, the addition of nuclease improved the efficiency of biofilm removal only at high concentrations, and no effect has been observed for *E. faecalis* biofilms. At pH 5.0, the protease-nuclease mixture appeared advantageous compared to solely enzymes at concentrations of 0.06%.

### 3.2. The effect of dishwashing liquids with nuclease and protease on rigid biofilms

Next, the biofilm-destruction ability of nuclease, protease and their mixture has been investigated in the composition of dishwashing liquid. Bacterial biofilms were prepared as described above and treated for 15 min with dishwashing liquid supplemented with either protease or nuclease or the protease-nuclease mixture (0.06% of each protein). Then the liquid was discarded, and residual biofilms were quantified with crystal violet staining.

**Figure 2.**
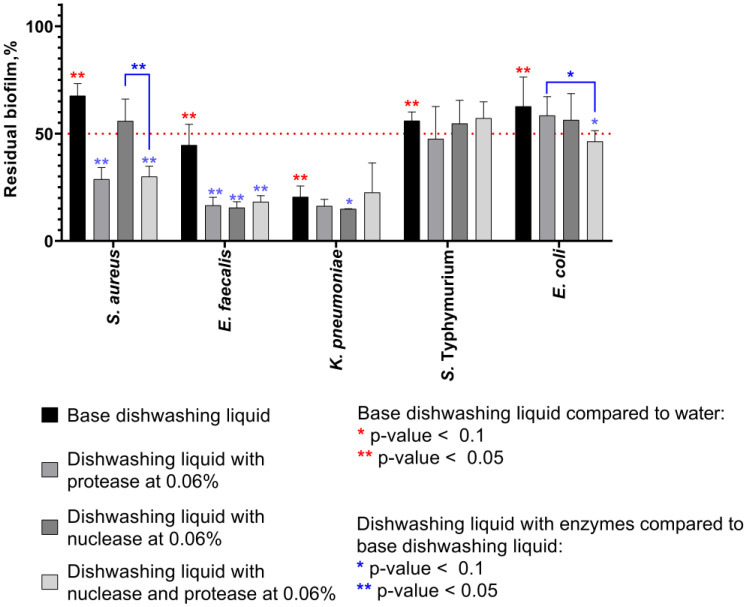
The destruction of biofilms of *S. aureus*, *E. faecalis*, *E. coli*, *S*. Typhimurium, and *K. pneumoniae* by 15 min treatment with dishwashing liquid base or liquids supplemented with both protease and nuclease (0.06% of each protein). 48-hour-old biofilms were treated for 15 min, and residual biofilms were quantified by CV staining. Averages and SDs are shown. The significance of differences between treated and untreated samples was assessed using the Multiple t-test with Holm-Sidak correction. Reds show significant differences between water-treated samples and samples treated with any dishwashing liquids; blues show significant differences between samples treated with dishwashing liquids with and without enzymes.

Figure 2 shows that the base dishwashing liquid itself was able to remove the rigid 48-h old biofilms, with the pronounced efficiency on *K. pneumoniae*, apparently because of the particular qualities of the biofilm structure formed by this bacterium [12],[47]. A 1.5-2-fold reduction of the biofilm biomass by the basic liquid treatment could be observed for other bacteria, although the presence of enzymes did not significantly improve the removal of rigid biofilms. As an exception, the dishwashing liquid containing both protease and nuclease was more effective in the destruction of *E. coli* biofilm.

### 3.3. The removal of adherent bacteria from dishes by the dishwashing liquids with nuclease and protease

In marked contrast to the model biofilms prepared in plates, dish surfaces have rather adherent bacteria than a real rigid biofilm. Therefore, we tested whether the supplementation of dishwashing liquids by nuclease and protease would increase the effectiveness of the removal of bacteria adhered to the surfaces of glass Petri dishes. To obtain adherent bacteria, suspensions of either *S. aureus*, *E. faecalis*, *K. pneumoniae*, *S*. Typhimurium, or *E. coli* with a density of 10^6^ CFU/mL were incubated in sterile Petri dishes for 2 hours at room temperature. Then the dishes were rinsed with sterile water, and filled with either dishwashing liquid with enzymes, sterile distilled water, or dishwashing liquid without enzymes for 15 min. Then the dishes were gently rinsed with sterile water, and remaining bacteria were quantified by both CFUs count and quantitative PCR.

**Figure 3.**
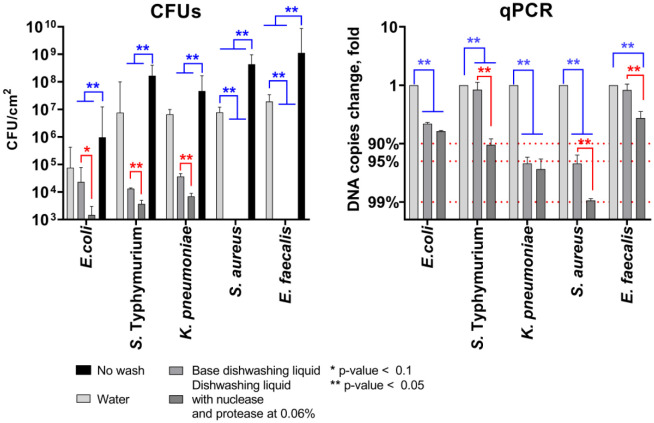
The removal of adherent bacteria from the surfaces of glass Petri dishes by 15 min. treatment with dishwashing liquid base or supplemented with both protease and nuclease (0.06% of each protein). Sterile Petri dishes were incubated for 2 h with bacterial suspension (10^9^ CFU/mL), rinsed with sterile water and then placed in either dishwashing liquid with enzymes, sterile distilled water, or dishwashing liquid base for 15 min., and after washing, remaining bacteria were quantified by CFU count (A, medians with the range are shown) and quantitative PCR (B, averages with SD are shown). The significance of differences was assessed using the Kruskal-Wallis test with Dunn's correction. **p < 0.05. Reds show significant differences between water-treated samples and samples treated with any dishwashing liquids; blues show significant differences between samples treated with dishwashing liquids with and without enzymes.

As can be seen from Figure 3, while the wash by the pure water allowed removal up to 90–99% of bacteria from the glass surface, the base dishwashing liquid provided more than a 3-log decrease of viable cells (excepting *E. coli*) with no detection of neither *S. aureus* nor *E. faecalis*, suggesting apparently their death. This assumption is partially confirmed by detection of bacterial DNA in amounts up to 100-fold lower, with no absolute absence. The supplementation by the protease-nuclease led to significantly deeper removal of Gram-negative bacteria as judged by CFUs counting. The quantitative PCR data matched the observed efficiency of the dishwashing liquid with enzymes as a significantly lower amount of bacterial DNA could be detected in samples swabbed from dishes washed by dishwashing liquid with enzymes compared to the base.

### 3.4. The removal of adherent bacteria from vegetables by the dishwashing liquids with nuclease and protease

Next, we tested whether the dishwashing liquids with nuclease and protease would be superior compared to the pure water and the base dishwashing liquid in bacterial removal from the surfaces of various vegetables. For that, iceberg lettuce, celery, cucumber, and the red apple were used to model vegetables with bacterial contamination on surfaces. To obtain adherent bacteria, fragments of leaves and peels were immersed in a suspension of either *S. aureus*, *E. faecalis, K. pneumoniae*, *S*. Typhimurium, or *E. coli* with a density of 10^6^ CFU/mL and incubated for 2 hours at room temperature, rinsed once with sterile water, and then placed in either sterile distilled water, dishwashing liquid without enzymes, or dishwashing liquid with enzymes for 15 min. Then the dishwashing liquids were washed from the samples, and the remaining bacteria were quantified by CFUs count.

**Figure 4.**
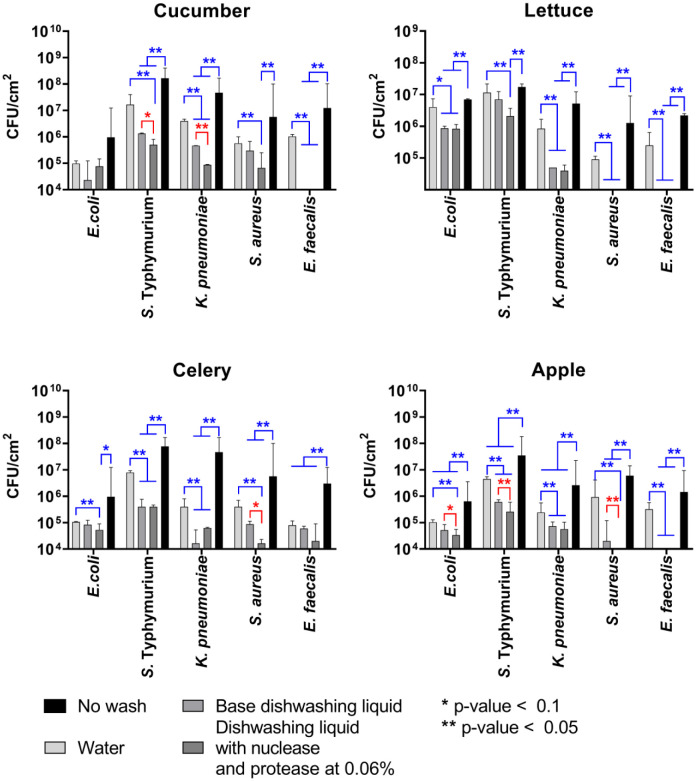
The removal of adherent bacteria from the surfaces of fruits and vegetables by 15 min. treatment with dishwashing liquid base or supplemented with both protease and nuclease (0.06% of each protein). Fragments of leaves and peels with adherent bacteria were rinsed with sterile water and then placed in either dishwashing liquid with enzymes, sterile distilled water, or dishwashing liquid without enzymes for 15 min., and after washing, remaining bacteria were quantified by CFU count. Medians with the range are shown. The significance of differences was assessed using the Kruskal-Wallis test with Dunn's correction. **p < 0.05. Reds show significant differences between water-treated samples and samples treated with any dishwashing liquids; blues show significant differences between samples treated with dishwashing liquids with and without enzymes.

Figure 4 indicates that bacteria were capable of adhering to all vegetables (up to 10^8^–10^9^ CFUs/cm²), and rinsing with tap water (without any mechanical action) significantly reduces the amount of CFUs only in the case of the apple peel. *S*. Typhimurium exhibited the highest adherence to all plant surfaces, and after the wash with water (light gray bars), up to 10^7^ CFUs/cm² could still be detected. The quantity of other bacteria was 10^5-7^ CFUs/cm². The wash with the base dishwashing liquid reduced the number of viable Gram-negative bacteria on the leaf surfaces by one order of magnitude compared to rinsing with pure water. This fits with previous reports that no significant differences in microbial populations were found on vegetables after decontamination with tap water, detergent, or benzalkonium chloride [48], which can be a consequence of increased tolerance of the adherent and biofilm-embedded bacteria to treatments [49],[50]. Thus, the number of CFUs of *S*. Typhimurium was significantly reduced when treated with base dishwashing liquid from celery and apple; the *E. coli* could not be removed. The supplementation of the base with enzymes allowed deeper removal of *S*. Typhimurium also from cucumber and lettuce, and *E. coli* from celery and apple. While *K. pneumoniae* could be washed out with the base liquid from all model vegetables, the presence of enzymes allowed 10-fold deeper removal from the cucumber. At the same time, the amount of *S. aureus* and *E. faecalis* CFUs could be significantly reduced by the base from lettuce and apple, the enzymes-supplemented base increased CFUs reduction rate 10-fold and more.

**Figure 5.**
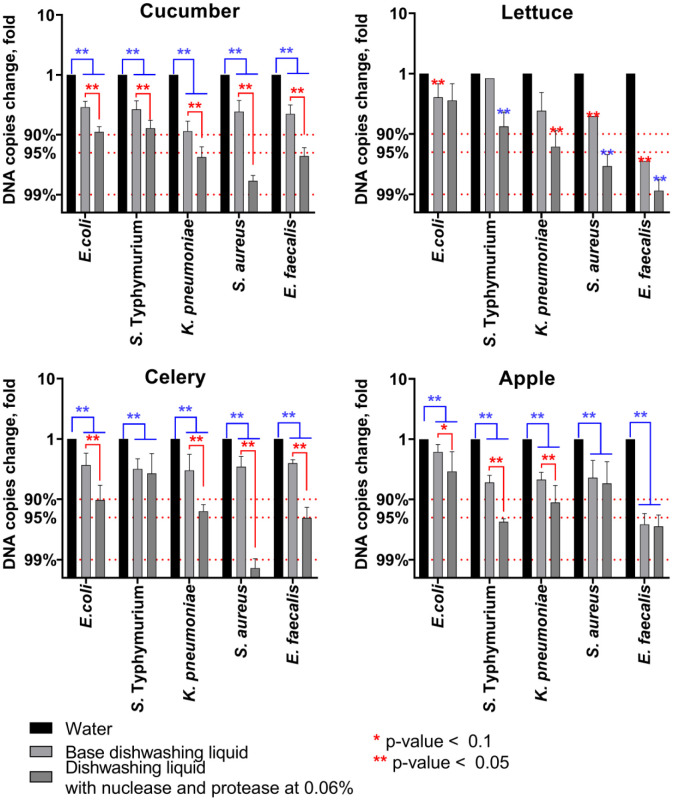
The removal of adherent bacteria from the surfaces of fruits and vegetables by 15 min treatment with dishwashing liquid base or supplemented with both protease and nuclease (0.06% of each protein). Fragments of leaves and peels with adherent bacteria were rinsed with sterile water and then placed in either dishwashing liquid with enzymes, sterile distilled water, or dishwashing liquid without enzymes for 15 min, and after washing, remaining bacteria were quantified by quantitative PCR. Averages with SD are shown. The significance of differences was assessed using the Kruskal-Wallis test with Dunn's correction. **p < 0.05. Reds show significant differences between water-treated samples and samples treated with any dishwashing liquids; blues show significant differences between samples treated with dishwashing liquids with and without enzymes.

Since the CFU counting has relatively low accuracy when the reduction rate is below 10 [46], the efficiency of bacterial cell removal has been checked with quantitative PCR. According to quantitative PCR data, the base dishwashing liquid provides a 2–8-fold reduction of bacterial DNA detectable on the leaves and peels (Figure 5), which in general fits with the CFU reduction rate (compare with Figure 4). The dishwashing liquid with enzymes demonstrated a significantly higher rate of bacterial DNA copy decrease, approximately by a factor of 2–8 compared with the basic dishwashing liquid and by a factor of up to 100-fold compared with water wash. Thus, on average, removal of up to 95–99% of the biomass for *S. aureus* and *E. faecalis*, as well as 90–95% for Gram-negative bacteria, could be observed (Figure 5).

Finally, the removal of adherent bacteria from leaves and peels with 1000-fold diluted dishwashing liquids, base or supplemented with both protease and nuclease (0.06% of each protein), has been tested (Figure S3). Expectedly, the bacterial removal efficiency reduced drastically. The dishwashing liquid base reduced the bacterial DNA copies on average by only 2-fold, while the addition of enzymes increased the reduction rate up to 4-8-fold, suggesting that the presence of hydrolytic enzymes in dishwashing liquids appears nevertheless beneficial for the removal of foodborne pathogens from vegetables and fruits.

## 4. Discussion

Significant consumption of unprocessed fruits and vegetables is among the most common nutrition requirements essential for human health, immunity, and overall well-being [51]. Nevertheless, plant surfaces typically contain various bacteria, including those leading to foodborne diseases, thus increasing the risks of foodborne infection contraction [1]–[3],[52]. Besides natural sources like soil and biofertilizers, the contamination of fresh-cut products often occurs during their transportation and storage, increasing the requirements of their decontamination prior to consumption. While rinsing off vegetables and fruits with simple tap water is widely recommended, reports of its efficiency for the removal of bacterial contamination from leaves and peels are somewhat contradictory. Thus, in several works it has been shown to be almost inefficient [48],[53] while, on the contrary, the reduction of the microbial seeding up to 100-fold has been reported [13],[14]. While in some works the use of disinfectants was offered to minimize the risk for consumers, their microbial reduction efficiency varies between 1 and 3 log10 CFU/g (for a recent review, we refer to [54], indicating only a limited disinfection effect). On the other hand, the remaining traces of disinfectants are also not health-friendly. Furthermore, no significant differences in microbial populations were found on vegetables after their decontamination with tap water, detergent, or benzalkonium chloride [48]. Moreover, many bacteria remain adherent (for a recent review, we refer to [15]), thus keeping a risk of food-borne infections, especially for children and persons with repressed immunity driven by the gut microbiota dysbiosis [16]–[20] Accordingly, finding alternative biocompatible and efficient solutions is of immense importance. Among them, enzymatic treatment is a perspective approach to the fresh-cut product decontamination in a common household environment [54].

In this work, we evaluated the potential of protease and nuclease (deoxyribonuclease) in a composition of dishwashing solution for the elimination of adherent cells of food-borne bacteria, like *S*. Typhimurium, *K. pneumoniae*, *E. coli*, *S. aureus*, and *E. faecalis*, from various fruits and vegetables (lettuce, cucumber, celery, and apple) and biofilms from the surfaces. Indeed, the dishwashing liquid containing enzymes provided a reduction in the number of CFUs of adherent bacteria from leaves and peels by 5–10 fold or greater compared to the base liquid, providing the removal of 90–99% of viable *S*. Typhimurium, *S. aureus*, and *E. faecalis* from green products (Figure 3), which fits well with the qPCR data (Figures 4 and 5). In general, the observed effectiveness of adherent *S. aureus* and *S*. Typhimurium removal from leaves and peels fits with the data obtained for these bacteria on the plate when the mature biofilm was treated with pure enzymes (Figure 1). Ass well, more efficient removal of Gram-negative bacteria with dishwashing liquid supplemented with both protease and nuclease could be achieved on glass dishes (Figure 3), while *S. aureus* and *E. faecalis* biofilms could be efficiently removed even by base dishwashing liquid. Unexpectedly, only half-reduction of the *K. pneumoniae* and *E. coli* has been observed in *in vitro* assays with either pure enzymes or dishwashing liquid with enzymes, apparently because their biofilm matrixes consist mainly of polysaccharides [47]. Furthermore, both dishwashing liquids base and supplemented with enzymes were of the same efficiency in the removal of the rigid two-days-old biofilms, that fits with earlier data [48],[53],[54]. Thus, while 2.5-fold increase in efficiency in the removal of *S. aureus* and *E. faecalis* biofilms has been detected compared to the tap water wash, any of dishwashing liquid were inefficient in the removal of biofilms formed by Gram-negative bacteria. The lowest efficiency of enzymatic treatment has been observed against *E. coli* in all models used in the study. While half-reduction of *E. coli* biofilm has been observed in *in vitro* assays after treatment with either pure enzymes or dishwashing liquid with enzymes, only a 10-fold reduction of adherent cells from the celery and cucumber surfaces could be achieved in the best case. Similar effectiveness of CFU removal was reported previously for the treatments with peracetic acid (100 ppm for 15 min) and hydrogen peroxide (133 ppm for 30 min) that reduced the total mesophilic microbial counts by about 2.8 log [48].

Specific molecular mechanisms of such selectivity to biofilms formed with Gram-positive and Gram-negative bacteria remain discussable. As a speculation, it could be attributed to the relatively high fraction of proteins in EPS of Gram-positive bacteria [29]. Thus, the proteins could be firstly denatured by surface-active components and thus become susceptible to proteases. Furthermore, the adhesins of Gram-positive microorganisms providing the attachment have a protein nature, and their structure could be disrupted by proteases [55],[56]. In marked contrast, the EPS of Gram-negative bacteria, consisting mainly of polysaccharides and nucleotides [47], can be covered with ionogenic surface-active components and thus close the EPS from the enzymes. Nevertheless, these assumptions remain speculative and require further investigations.

## 5. Conclusions

Taken together, our data clearly indicate that protease, both individually and especially in mixture with nuclease, is an attractive additive to dishwashing liquids, representing a biocompatible, efficient, and thus altogether promising solution for decontaminating the surfaces of fruits and vegetables from adherent bacteria and bacterial biofilms. Among the compositions tested, the system with a protease and nuclease at 0.06% of each demonstrates promising effectiveness. The most pronounced improvement has been observed against Gram-positive bacteria, providing removal of up to 99% of cells, while up to 90-95% of Gram-negative bacteria could also be removed, compared to only a 2–5 fold reduction of adherent cells when solely dishwashing liquids without enzymes were used.

## Use of AI tools declaration

The authors declare they have not used Artificial Intelligence (AI) tools in the creation of this article.


