# Supplementary material for: Dishwashing liquids with nuclease and protease: An improved biocompatible solution for the removal of adherent bacteria from fruits and vegetables
Source: AIMS Microbiol. 2025 Dec 29;11(4):1101–18. doi: 10.3934/microbiol.2025048 (PMC12782945; doi:10.3934/microbiol.2025048)
Supplement: Supplementary file 1 [file microbiol-11-04-048-s001.pdf]

AIMS Microbiology

AIMS Microbiology, 11(4): 1101–1118.
DOI: 10.3934/microbiol.2025048
Received: 02 September 2025
Revised: 21 December 2025
Accepted: 26 December 2025
Published: 29 December 2025

https://www.aimspress.com/journal/microbiology

Research article

Dishwashing liquids with nuclease and protease: An improved biocompatible solution for the removal of adherent bacteria from fruits and vegetables

Lyudmila Ayzatullina[1], Sofia Kolyshkina[1], Elizaveta Patronova[2], Viktor Filatov[3], Iva Zadorina[1], Maya Kharitonova[1], Mikhail Bogachev[4] and Airat Kayumov[1,*]

[1] Institute of Fundamental Medicine and Biology, Kazan Federal University, Kazan, Russian Federation

[2] Institute of Biomedical Systems and Biotechnology, Peter the Great St.Petersburg Polytechnic University, Saint Petersburg, Russian Federation

[3] Department of Pharmaceutical Chemistry and Organization of Pharmaceutical Business, Faculty of Fundamental Medicine, Lomonosov Moscow State University, Moscow, Russian Federation

[4] Biomedical Engineering Research Centre, St. Petersburg Electrotechnical University, St. Petersburg, Russian Federation

* **Correspondence:** Email: kairatr@yandex.ru.

Supplementary

Table S1. Base dishwashing liquid detergent.

Compound	Content, weight %
Water purified	u
Sodium Laureth Sulfate	16.0
C8-10 Fatty Alcohol Glycoside	12.0
Cocamidopropyl betaine	2.0
Glycerin	1.0
salt	0.3
pH regulator (citric acid monohydrate or sodium hydroxide)	0.5
Combined preservative (sodium benzoate, potassium sorbate)	1.1

Table S2. Dishwashing liquid detergent with enzymes.

Compound	Content, weight %
Water purified	u
Sodium Laureth Sulfate	16.0
C8-10 Fatty Alcohol Glycoside	12.0
Cocamidopropyl betaine	2.0
Nuclease (DNAse)	.
Protease sublitisin	0.06-0.25
Glycerin	1.0
salt	0.3
pH regulator (citric acid monohydrate or sodium hydroxide)	0.5
Combined preservative (sodium benzoate, potassium sorbate)	1.1

Table S3. Dishwashing liquid detergent with enzymes.

Compound	Content, weight %
Water purified	u
Sodium Laureth Sulfate	16.0
C8-10 Fatty Alcohol Glycoside	12.0
Cocamidopropyl betaine	2.0
Nuclease (DNAse)	.
Glycerin	1.0
salt	0.3
pH regulator (citric acid monohydrate or sodium hydroxide)	0.5
Combined preservative (sodium benzoate, potassium sorbate)	1.1

. Dishwashing liquid detergent with enzymes.

Compound	Content, weight %
Water purified	u
Sodium Laureth Sulfate	16.0
C8-10 Fatty Alcohol Glycoside	12.0
Cocamidopropyl betaine	2.0
Nuclease (DNAse)	.
Protease sublitisin	0.06-0.25
Glycerin	1.0
salt	0.3
pH regulator (citric acid monohydrate or sodium hydroxide)	0.5
Combined preservative (sodium benzoate, potassium sorbate)	1.1

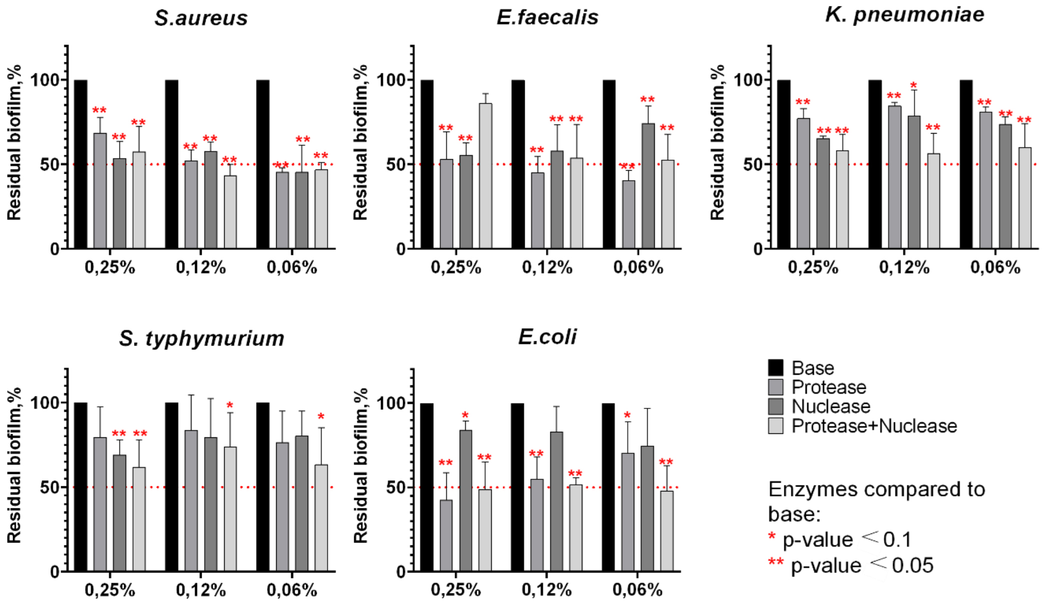

Figure S1. The destruction of biofilms of *S. aureus, E. faecalis, E. coli, S. typhimurium* and *K. pneumoniae* by either protease, nuclease, (0.06, 0.12 and 0.25 0.5 %) or the protease-nuclease mixture (0.06, 0.12 and 0.25 % of each protein) in PBS with pH 6.0. A 48 h old biofilms were treated for 15 min with enzymes and residual biofilms were quantified by CV-staining. Averages and SDs are shown. The significance of differences between treated and untreated samples was assessed using Kruskal-Wallis test with Holm-Sidak correction.

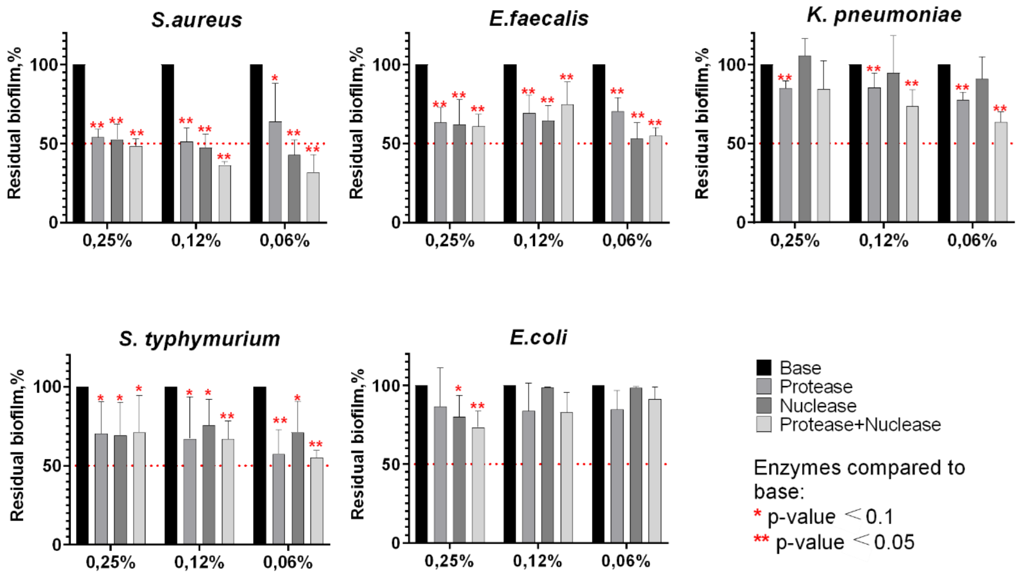

Figure S2. The destruction of biofilms of *S. aureus, E. faecalis, E. coli, S. typhimurium* and *K. pneumoniae* by either protease, nuclease, (0.06, 0.12 and 0.25 0.5 %) or the protease-nuclease mixture (0.06, 0.12 and 0.25 % of each protein) in PBS with pH 5.0. A 48 h old biofilms were treated for 15 min with enzymes and residual biofilms were quantified by CV-staining. Averages and SDs are shown. The significance of differences between treated and untreated samples was assessed using Kruskal-Wallis test with Holm-Sidak correction.

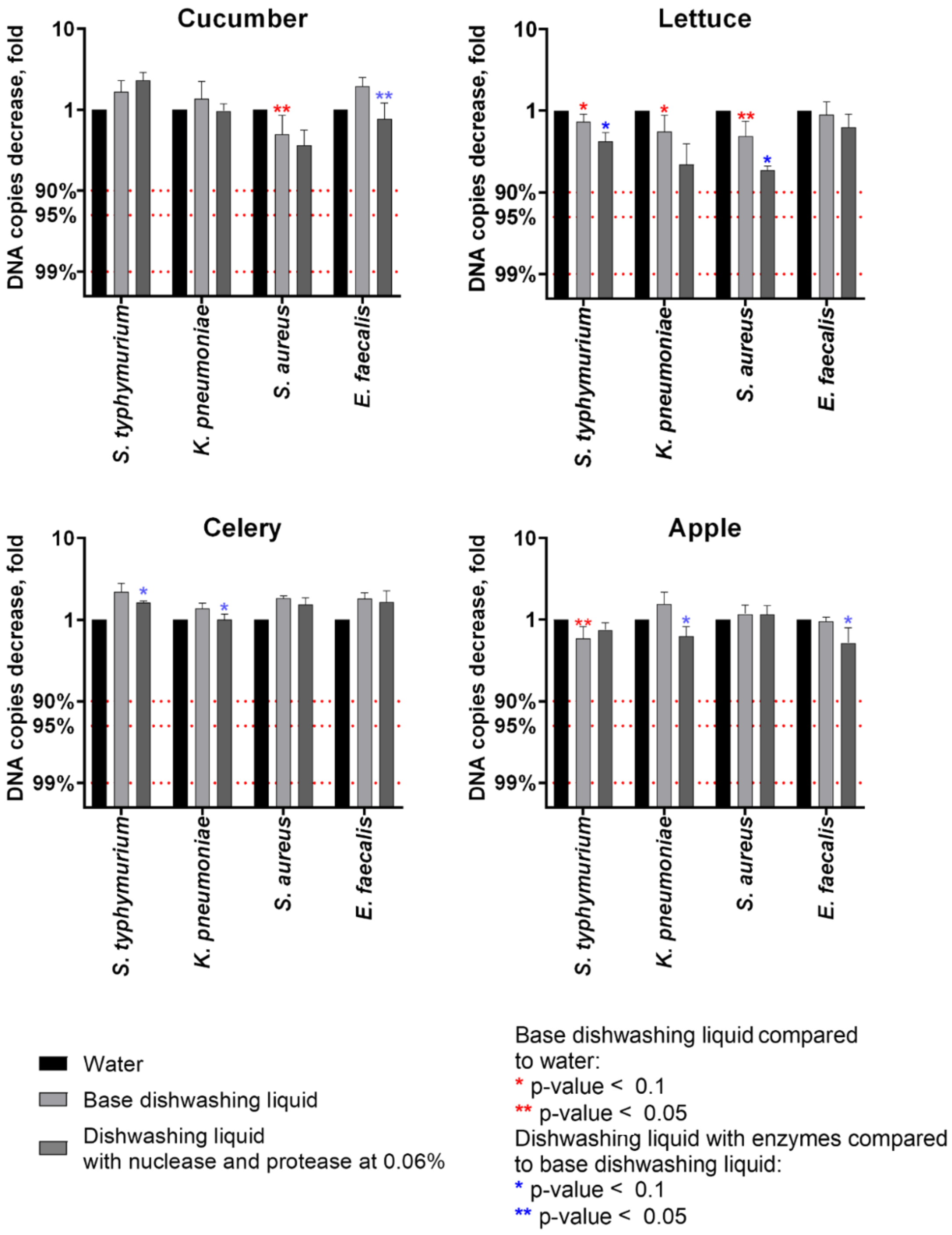

Figure S3. The removal of adherent bacteria from the surfaces of fruits and vegetables by 15 min treatment with 1000-fold diluted dishwashing liquids base or supplemented with both protease and nuclease (0.06% of each protein). Fragments of leafs and peels with adherent bacteria were rinsed by sterile water and then placed in either dishwashing liquid with enzymes, sterile distilled water or the dishwashing liquid without enzymes for 15 min and after washing remaining bacteria were quantified by quantitative RT-PCR. Averages with SD are shown. The significance of differences was assessed using Kruskal-Wallis test. **p<0.05. Reds show significant differences between water-treated samples and samples treated with

any dishwashing liquids, blues show significant differences between samples treated with dishwashing liquids with and without enzymes.

AIMS Press

© 2025 the Author(s), licensee AIMS Press. This is an open access article distributed under the terms of the Creative Commons Attribution License (https://creativecommons.org/licenses/by/4.0)
